# Isolation, Identification, Virulence and Pathogenic Features of *Lactococcus garvieae* from Cage-Cultured Tilapia (*Oreochromis niloticus*) in Thailand

**DOI:** 10.3390/ijms27083469

**Published:** 2026-04-13

**Authors:** Yosapon Adisornprasert, Benchawan Kumwan, Pakapon Meachasompop, Chonlatat Rajitdumrong, Pimrawee Chaemlek, Prapansak Srisapoome, Wararut Buncharoen, Natthapong Paankhao, Niyada Umputhorn, Chonthicha Choppradit, Pichasit Sangmek, Sittichai Hatachote, Putita Chokmangmeepisarn, Kednapat Sriphairoj, Anurak Uchuwittayakul

**Affiliations:** 1 Center of Excellence in Aquatic Animal Health Management, Faculty of Fisheries, Kasetsart University, Bangkok 10900, Thailand; yosapon.ad@ku.th (Y.A.); benchawan.kumw@ku.th (B.K.); pakapon.meac@ku.th (P.M.); chonlatat.ra@ku.th (C.R.); pimrawee.ch@ku.th (P.C.); ffispssp@ku.ac.th (P.S.); 2 Laboratory of Aquatic Animal Health Management, Department of Aquaculture, Faculty of Fisheries, Kasetsart University, Bangkok 10900, Thailand; 3 Special Research Incubator Unit for Development and Application of Vaccine Delivery Systems for Aquatic Animals, Department of Aquaculture, Faculty of Fisheries, Kasetsart University, Bangkok 10900, Thailand; 4 Department of Biology, Faculty of Science, Chiang Mai University, Chiang Mai 50200, Thailand; wararut.bun@cmu.ac.th; 5 Kamphaeng Saen Fisheries Research Station, Faculty of Fisheries, Kasetsart University, Kamphaeng Saen Campus, Nakhon Pathom 73140, Thailand; ffisnpp@ku.ac.th; 6 Faculty of Natural Resources and Agro-Industry, Kasetsart University, Chalermphrakiat Sakon Nakhon Province Campus, Sakon Nakhon 47000, Thailand; niyadaum@gmail.com (N.U.); chonticha.020428@gmail.com (C.C.); pichasit.sa@ku.th (P.S.); sittichai.h@ku.th (S.H.); 7 Department of Microbiology, Faculty of Science, Kasetsart University, Bangkok 10900, Thailand; putita.cho@ku.th

**Keywords:** lactococcosis, *Lactococcus garvieae*, Nile tilapia, cage culture, reservoir aquaculture, tissue microbiome, blood diagnosis, LD_50_, LC_50_, challenge trial, histopathology, Thailand

## Abstract

Lactococcosis caused by *Lactococcus garvieae* is an emerging threat to warmwater aquaculture, yet evidence integrating field outbreaks with robust molecular confirmation and controlled virulence testing remains limited for Thailand’s cage-cultured tilapia. From May to October 2025, acute mortality events were investigated in cage-cultured Nile tilapia (*Oreochromis niloticus*) in a reservoir in Ubon Ratchathani Province, Thailand. Suspected outbreaks were defined by abrupt daily mortality exceeding 5% accompanied by septicemia-like clinical signs. Water quality during sampling covered the following ranges: temperature 28.6–31.9 °C, pH 6.5–7.0, salinity 0.02–0.03 ppt, electrical conductivity 0.036–0.046 mS/cm, TDS 22.20–26.50 mg/L, total alkalinity 17.0–34.0 mg/L as CaCO_3_, total hardness 12.0–60.0 mg/L as CaCO_3_, dissolved oxygen 6.5–7.0 mg/L, and NH_3_ were below the limit of detection. Full-length *16S rRNA* tissue profiling revealed strong tissue partitioning: blood microbiomes were consistently dominated by *Lactococcus* and *L. garvieae* at the species level, whereas gills showed higher richness and mixed communities with multiple opportunistic taxa. Culture isolation was more reliable from blood than gills, yielding 16 Gram-positive, catalase-negative isolates (AAHM-LG2501–AAHM-LG2516) that clustered within the *L. garvieae* clade in near full-length *16S rRNA* phylogenetic analysis and were separated from closely related *Lactococcus* lineages. A representative blood isolate (AAHM-LG2501) showed dose-dependent virulence in controlled challenges, with an LD_50_ of ~1.05 × 10^5^ CFU/fish by intraperitoneal injection and an LC_50_ of ~1.20 × 10^6^ CFU/mL by immersion. Histopathology supported systemic dissemination, with injection producing more consistent multi-organ lesions than immersion, particularly in head kidney, liver, and spleen, while gills exhibited route-associated epithelial and vascular alterations. Together, these findings confirm *L. garvieae* as a major etiological agent of septicemic outbreaks in cage-cultured tilapia in Thailand and support a practical surveillance framework prioritizing blood sampling, molecular confirmation, and risk-based monitoring to guide biosecurity and vaccine-oriented prevention.

## 1. Introduction

*Lactococcus garvieae* causes lactococcosis, a rapidly progressive septicemic disease that threatens warmwater aquaculture worldwide. In tilapia farming, outbreaks are often linked to elevated water temperatures and intensive production, conditions that are common in Thailand’s cage-culture systems. Cages concentrate biomass in open water, increase contact rates among fish, and expose stocks to waterborne pathogens moving through shared hydrological networks within farms and between neighbors [1,2].

Clinically, tilapia with lactococcosis often show early, nonspecific signs such as decreased appetite, lethargy, abnormal swimming, and skin darkening. Sudden mortality has been reported in red tilapia (*Oreochromis* spp.), with clinical features resembling streptococcal infections. As disease advances, fish frequently develop unilateral or bilateral exophthalmia, abdominal distension, and occasional anal prolapse [2,3,4]. Necropsy commonly reveals ascites, hepatosplenomegaly, and serosal petechiae. These signs overlap with other bacterial diseases of tilapia, so standardized clinical recording and targeted sampling are essential [1]. In cage settings, environmental stressors, fluctuating dissolved oxygen, and organic loading can predispose to outbreaks and may modify lesion profiles. Co-infections, particularly with Gram-negative bacteria such as *Aeromonas* spp., can intensify pathology and complicate case management, underscoring the value of multiplex diagnostic panels during investigations [1,5,6].

Virulence in *L. garvieae* is multifactorial. Capsulation is a well-recognized determinant that enhances resistance to phagocytosis and correlates with severe disease. Surface adhesins facilitate colonization of mucosal tissues, and hemolysins contribute to cytolysis and vascular injury, aligning with field pathology [7,8]. Antimicrobial susceptibility patterns vary among regions and over time. Some isolates remain broadly susceptible, while others display resistance to multiple agents, likely reflecting antimicrobial usage histories and gene flow. Empirical therapy without local susceptibility testing risks treatment failure and the selection of resistant strains [9,10].

Epidemiologically, outbreaks are associated with warm seasons, high stocking densities, and rapid fish movement across the supply chain. In cage-culture landscapes, hydrological connectivity allows pathogens to traverse cages and farms, while shared equipment and boats can act as fomites. Genotyping has revealed population structure and clonal dissemination within regions, information that can guide containment [2,5,6,7,11,12]. Spatiotemporal analytics integrating farm records, water parameters, fish movements, and molecular data can identify hotspots, reveal transmission corridors, and quantify risk factors such as temperature thresholds and stocking practices. Such analyses are particularly relevant for Thailand, where large-scale tilapia cage production intersects with monsoonal hydrology and seasonal thermal peaks.

This study characterizes the clinical, molecular, and epidemiological features of *Lactococcus garvieae* in cage-cultured tilapia in Thailand, addresses critical gaps by integrating three domains. First, we standardize field clinical characterization, linking signs and lesions to laboratory confirmation. Second, we apply a validated molecular workflow to ensure accurate species identification and assess genetic relatedness among isolates. Third, we conduct spatiotemporal analyses that merge molecular profiles with environmental and management data to identify clusters, infer transmission, and quantify risk factors. The expected outputs are a refined diagnostic pathway for frontline laboratories, risk-based surveillance markers for farms, and actionable biosecurity guidance tailored to Thailand’s cage-culture context. Collectively, these outcomes aim to reduce disease burden, improve production stability, and contribute regional evidence to the broader understanding of *L. garvieae* in warmwater aquaculture.

## 2. Results

### 2.1. Field Case Identification and Outbreak Presentation

From May to October 2025, disease investigations were conducted during acute mortality events affecting cage-cultured Nile tilapia in a large reservoir in Ubon Ratchathani Province, Thailand. Fish were reared in floating net cages (each 5 m × 5 m, 2.5 m depth) stocked at approximately 1000 fish per cage, and sampled fish typically ranged from 200 to 900 g. Mortality events occurred in multiple cages within the same farming area, consistent with a rapid, outbreak-like pattern in open-water culture systems.

**Figure 1:**
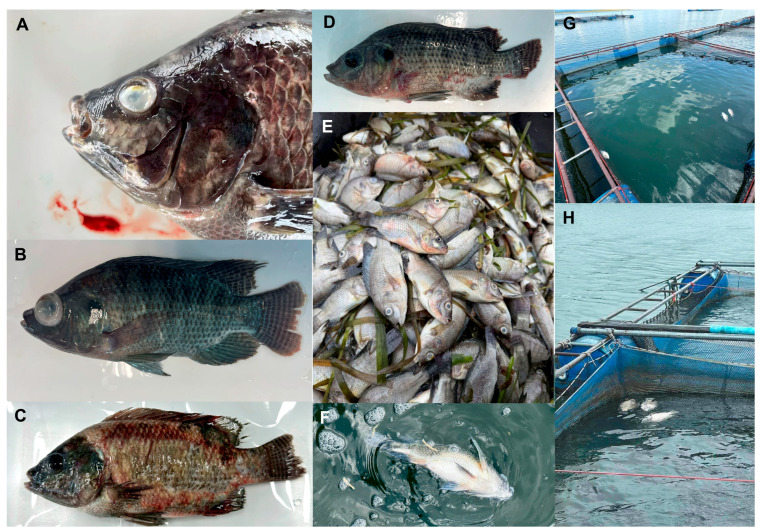
Clinical signs and field mortality associated with disease outbreaks in cage-cultured Nile tilapia (*Oreochromis niloticus*). (**A**) Close-up of an affected fish showing severe ocular opacity/exophthalmia and hemorrhagic changes around the head region. (**B**) Whole-body view of a moribund fish exhibiting abnormal coloration and poor condition. (**C**) Affected fish with extensive external lesions and hemorrhagic areas along the body surface. (**D**) Representative diseased fish displaying gross signs consistent with systemic bacterial infection. (**E**) High cumulative mortality was observed during an outbreak event. (**F**) Recently dead fish floating at the water surface. (**G**,**H**) Cage-culture setting in a reservoir and net-cage units where mortality events occurred, including dead fish accumulated within cages.

Suspected cases were identified when a cage showed an abrupt rise in daily mortality exceeding the operational threshold (greater than 5% on one or more days). During these events, affected fish commonly displayed lethargy, reduced feeding response, and marked body darkening, and a proportion of fish showed exophthalmia and abnormal behavior compatible with neurologic involvement. Gross examination frequently revealed external hemorrhagic changes on the skin and around fin bases, and severely affected fish were often moribund at the cage edge or found freshly dead in the net pens. These clinical and gross signs were consistent with a systemic bacterial septicemia-like syndrome observed during the mortality episodes (Figure 1).

### 2.2. Environmental Metadata During Mortality Events

Across the monitoring period (May to October 2025), lactococcosis-associated mortality was observed under the following water quality ranges: temperature 28.6–31.9 °C, pH 6.5–7.0, salinity 0.02–0.03 ppt, electrical conductivity 0.036–0.046 mS/cm, TDS 22.20–26.50 mg/L, total alkalinity 17.0–34.0 mg/L as CaCO_3_, total hardness 12.0–60.0 mg/L as CaCO_3_, and dissolved oxygen 6.5–7.0 mg/L and ammonia (NH_3_) were below the limit of detection (<LOD).

### 2.3. Tissue Distribution of the Causative Bacterium in Blood and Gills of Infected Fish

**Figure 2:**
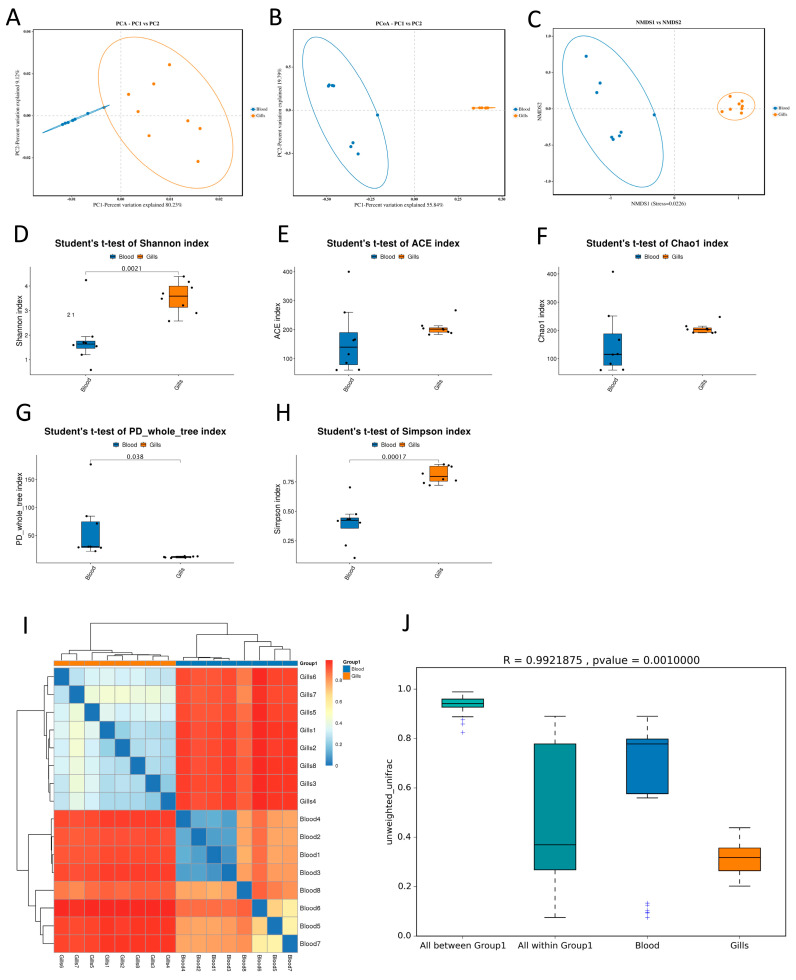
Beta-diversity, alpha-and NMDS) comparing microbial community structure between blood and gill diversity, and community separation between blood and gill microbiota. (**A**–**C**) Ordination analyses (PCA/PCoA samples. (**D**–**H**) Alpha-diversity indices (Shannon, ACE, Chao1, PD whole tree, and Simpson) compared between tissues using Student’s *t*-test. (**I**) Heatmap of sample-to-sample distances with hierarchical clustering showing tissue-associated grouping. (**J**) Unweighted UniFrac distance comparison demonstrating significant separation between blood and gill communities (R = 0.9921875, *p* = 0.001).

Beta-diversity analyses showed a clear separation of bacterial communities between blood and gill samples. Ordination plots (PCA/PCoA and NMDS) consistently clustered blood samples apart from gill samples, indicating distinct community structures by tissue (Figure 2A–C).

Alpha-diversity differed significantly between tissues (Figure 2D–H). Gill microbiota exhibited higher richness and evenness as reflected by significantly greater Shannon, ACE, Chao1, and Simpson indices compared with blood, whereas phylogenetic diversity (PD whole tree) was higher in blood than in gills.

Hierarchical clustering and distance-based comparisons further supported strong tissue-specific partitioning. The sample-to-sample heatmap demonstrated clustering primarily by tissue type (Figure 2I). Unweighted UniFrac distances also differed markedly between groups, showing strong community dissimilarity between blood and gills (R = 0.9921875, *p* = 0.001) (Figure 2J).

**Figure 3:**
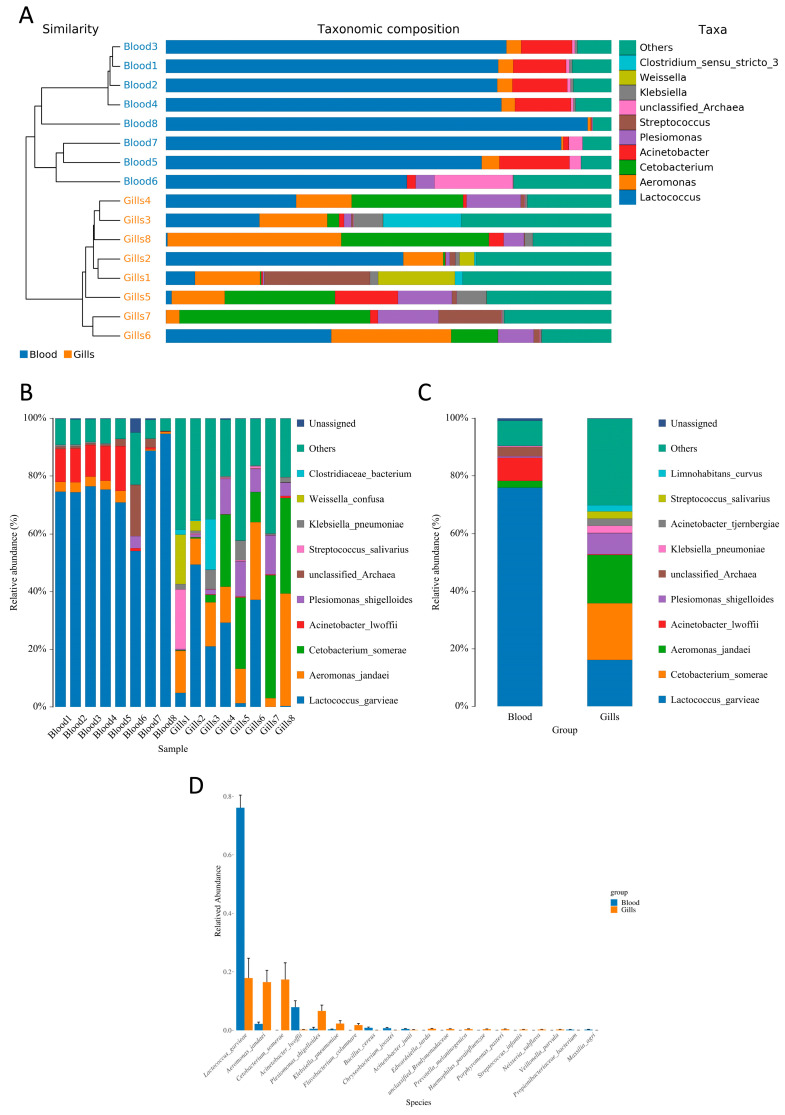
Taxonomic composition of blood and gill microbiota. (**A**) Hierarchical clustering (similarity) and stacked bar plots showing the relative abundance of major taxa across individual blood and gill samples. (**B**) Relative species abundance of dominant taxa at the sample level. (**C**) Group-level mean relative abundance comparing blood versus gills. (**D**) Species-level relative abundance comparison highlighting tissue-associated dominant species and differential contributions between blood and gills.

Taxonomic profiling demonstrated strong tissue-associated compositional shifts, with markedly reduced complexity in blood relative to gills (Figure 3). In the hierarchical clustering and stacked bar plots (Figure 3A), all blood samples (Blood1–Blood8) clustered together and were overwhelmingly dominated by the genus Lactococcus, whereas gill samples formed a distinct cluster and showed a broader mixture of taxa. In contrast to blood, gill communities contained substantial contributions from *Aeromonas*, *Cetobacterium*, *Acinetobacter*, *Plesiomonas*, *Streptococcus*, *Klebsiella*, and *Clostridium*, together with a larger “Others” fraction, reflecting higher community complexity and inter-individual variability in the gill niche (Figure 3A–C).

At the species level, the sample-wise profiles (Figure 3B) showed that *Lactococcus garvieae* constituted the dominant signal across all blood samples, while gill samples displayed mixed dominance patterns with prominent representation of *Aeromonas jandaei*, *Cetobacterium somerae*, *Acinetobacter lwoffii*, *Plesiomonas shigelloides*, and occasional *Klebsiella pneumoniae* and *Streptococcus salivarius*. Group-level averaging (Figure 3C) reinforced this contrast: blood was essentially characterized by *L. garvieae*, whereas the gill group represented a composite community where multiple bacterial species contributed meaningful proportions rather than a single dominant taxon.

Consistent with these patterns, the comparative species-enrichment plot (Figure 3D) highlighted *L. garvieae* as strongly enriched in blood, while gills showed higher relative contributions from several opportunistic or environmental-associated bacteria, including *A. jandaei*, *C. somerae*, *P. shigelloides*, *K. pneumoniae*, *Flavobacterium columnare*, *Bacillus cereus*, and *Chryseobacterium johnsonii*, alongside low-abundance detections of additional taxa (e.g., *Edwardsiella tarda*, *Prevotella melaninogenica*, *Haemophilus parainfluenzae*, *Porphyromonas pasteri*, *Streptococcus infantis*, *Neisseria shigella*, *Veillonella parvula*, *Propionibacteriaceae* bacterium, and *Massilia agri*) (Figure 3D).

### 2.4. Isolation and Identification of Causative Bacterium in Blood and Gills of Infected Fish

**Figure 4:**
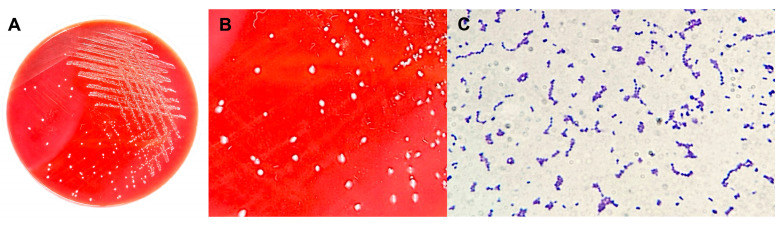
Isolation and preliminary phenotypic characterization of *Lactococcus garvieae* from diseased Nile tilapia. (**A**) Representative growth of presumptive *Lactococcus* colonies on tryptic soy blood agar (TSBA; 5% sheep blood) after incubation at 28–30 °C for 24–48 h. (**B**) Close-up view showing small, creamy-white colonies on blood agar with a consistent hemolysis pattern. (**C**) Gram-stained smear of a representative isolate showing Gram-positive cocci, typically arranged in pairs and short chains, consistent with *Lactococcus* spp.

A Gram-positive coccus consistent with *Lactococcus* spp. was successfully isolated from clinically infected fish (Figure 4). Isolation was more consistently achieved from blood samples (Blood1–Blood8; AAHM-LG2501–AAHM-LG2508) than from gill samples (Gill1–Gill8; AAHM-LG2509–AAHM-LG2516). Colonies on TSBA typically showed small, creamy-white growth (Figure 4A) with a characteristic hemolysis pattern (Figure 4B), and presumptive isolates were Gram-positive (Figure 4C), catalase-negative, oxidase-negative, and non-motile, supporting preliminary classification as lactic acid bacteria. Pure cultures were obtained after repeated sub-culturing and were preserved at −80 °C for downstream confirmation.

**Figure 5:**
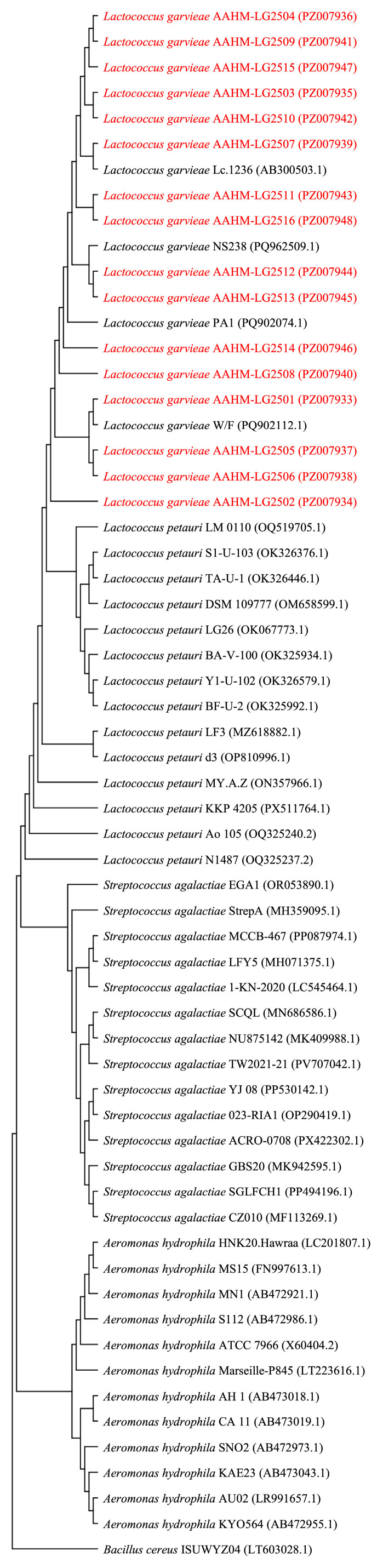
Phylogenetic confirmation of *L. garvieae* isolates based on near full-length 16S rRNA sequences. A phylogenetic tree was constructed using near full-length *16S rRNA* gene sequences from the study isolates (AAHM-LG2501 to AAHM-LG2516; accession numbers PZ007933-PZ007948, respectively) and reference sequences of *Lactococcus* spp. retrieved from public databases. Study isolates clustered within the *L. garvieae* clade alongside reference *L. garvieae* strains, and were clearly separated from the *Lactococcus petauri* clade. Additional reference taxa (*Streptococcus agalactiae*, *Aeromonas hydrophila*, and *Bacillus cereus*) were included to provide phylogenetic context.

Phylogenetic analysis based on near full-length 16S rRNA sequences supported the species assignment and tissue-associated findings. In the 16S rRNA tree, all study isolates (AAHM-LG2501 to AAHM-LG2516) clustered within the *L. garvieae* clade together with reference *L. garvieae* sequences (e.g., Lc.1236, NS238, PA1, W/F), confirming that the recovered field isolates belong to the *L. garvieae* lineage. Importantly, the *L. garvieae* cluster was clearly separated from a distinct *Lactococcus petauri* clade composed of multiple reference strains (e.g., LM 0110, DSM 109777, LG26, LF3, N1487), reducing the likelihood that the isolates were misassigned to closely related *Lactococcus* spp. based on 16S rRNA similarity alone. Additional bacterial taxa included in the tree (e.g., *Streptococcus agalactiae*, *Aeromonas hydrophila*, and *Bacillus cereus*) formed distant, well-separated branches, providing broader phylogenetic context and supporting correct placement of the *Lactococcus* lineages within the overall topology (Figure 5).

### 2.5. Determination of LD_50_ and LC_50_ for L. garvieae Challenge in Nile Tilapia

**Figure 6:**
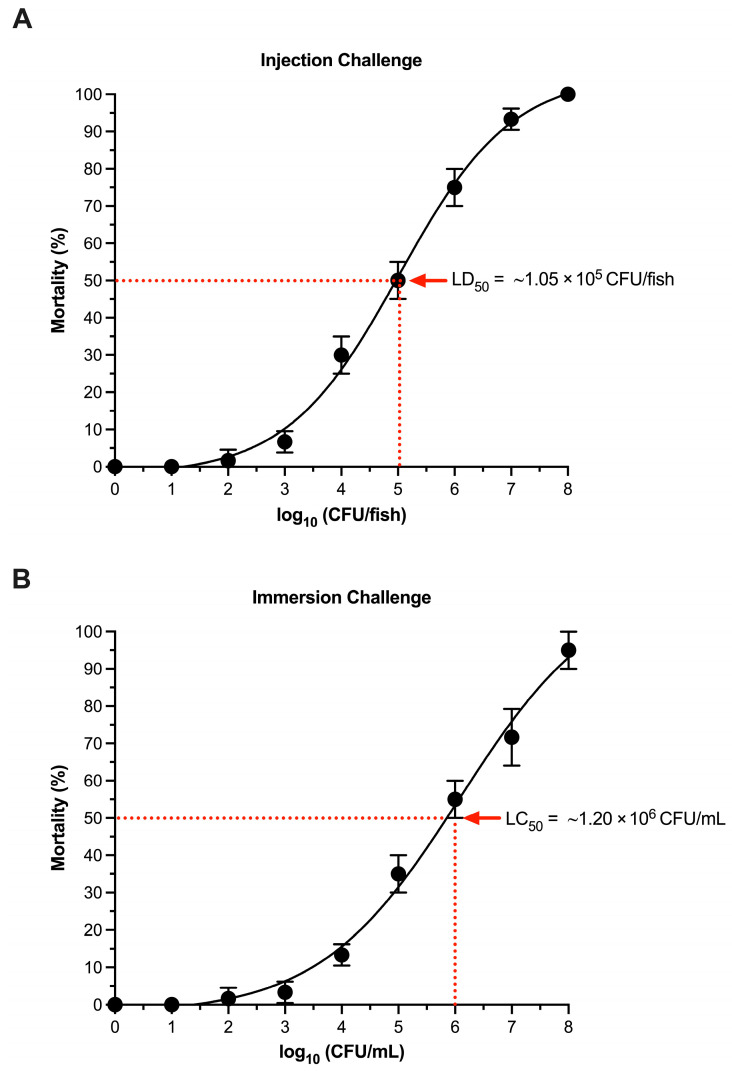
Dose–response estimation of LD_50_ and LC_50_ for *Lactococcus garvieae* AAHM-LG2501 in Nile tilapia. (**A**) Intraperitoneal injection challenge. Cumulative mortality (%) was plotted against log10-transformed dose (CFU/fish) and the dashed reference lines indicate the LD_50_ = ~1.05 × 10^5^ CFU/fish (50% predicted mortality). (**B**) Immersion challenge. Cumulative mortality (%) was plotted against log10-transformed bacterial concentration (CFU/mL) and dashed reference lines indicate the LC_50_ = ~1.20 × 10^6^ CFU/mL. (*n* = 20 fish per tank; three replicate tanks per treatment).

Dose–response curves showed a clear, monotonic increase in cumulative mortality with increasing *L. garvieae* challenge dose (injection) or concentration (immersion). Probit regression analysis estimated the median lethal dose (LD_50_) for intraperitoneal injection at 1.05 × 10^5^ CFU/fish, resulting in 50% predicted mortality (Figure 6A). Similarly, the immersion challenge produced a dose-dependent mortality response, and probit regression estimated the median lethal concentration (LC_50_) at 1.20 × 10^6^ CFU/mL, corresponding to the concentration predicted to cause 50% mortality under the immersion exposure model (Figure 6B).

### 2.6. Blood Biochemistry Profiles

**Figure 7:**
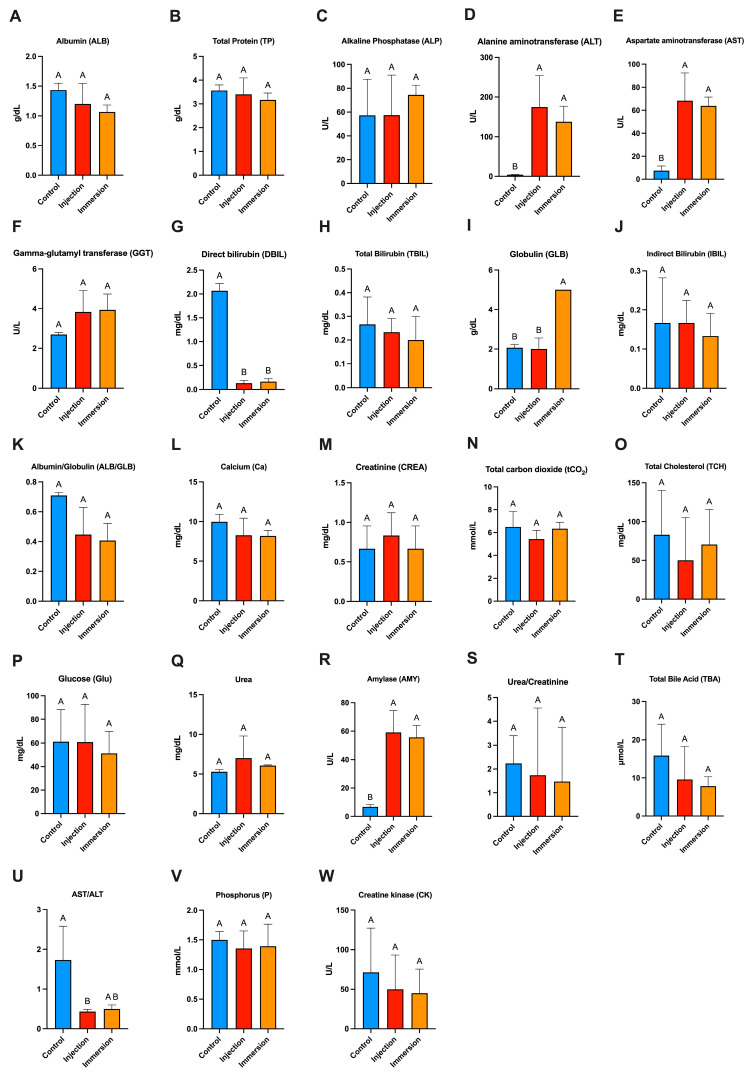
Blood biochemistry profiles of Nile tilapia following experimental challenge. Serum biochemical indices were compared among Control, Injection, and Immersion groups: (**A**) albumin (ALB), (**B**) total protein (TP), (**C**) alkaline phosphatase (ALP), (**D**) alanine aminotransferase (ALT), (**E**) aspartate aminotransferase (AST), (**F**) gamma-glutamyl transferase (GGT), (**G**) direct bilirubin (DBIL), (**H**) total bilirubin (TBIL), (**I**) globulin (GLB), (**J**) indirect bilirubin (IBIL), (**K**) albumin/globulin ratio (ALB/GLB), (**L**) calcium (Ca), (**M**) creatinine (CREA), (**N**) total carbon dioxide (tCO_2_), (**O**) total cholesterol (TCH), (**P**) glucose (Glu), (**Q**) urea, (**R**) amylase (AMY), (**S**) urea/creatinine ratio, (**T**) total bile acid (TBA), (**U**) AST/ALT ratio, (**V**) phosphorus (P), and (**W**) creatine kinase (CK). Bars represent mean ± SD. Different letters above bars indicate significant differences among groups (*p* < 0.05).

Blood biochemistry profiles differed modestly between groups, with most indices remaining comparable across control, injection, and immersion treatments (Figure 7A–W). Serum albumin (ALB), total protein (TP), alkaline phosphatase (ALP), gamma-glutamyl transferase (GGT), total bilirubin (TBIL), indirect bilirubin (IBIL), albumin/globulin ratio (ALB/GLB), calcium (Ca), creatinine (CREA), total carbon dioxide (tCO2), total cholesterol (TCH), glucose (Glu), urea, phosphorus (P), creatine kinase (CK), urea/creatinine ratio, and total bile acid (TBA) showed no significant differences among groups (*p* > 0.05).

In contrast, liver-associated enzymes and selected protein fractions showed significant changes following challenge. Alanine aminotransferase (ALT) and aspartate aminotransferase (AST) were significantly elevated in both injection and immersion groups relative to controls (*p* < 0.05), indicating increased hepatocellular or systemic tissue disturbance after bacterial exposure. Amylase (AMY) was also significantly higher in challenged fish (injection and immersion) compared with controls (*p* < 0.05).

Bilirubin fractions and globulin showed tissue-route-associated differences. Direct bilirubin (DBIL) was significantly higher in controls than in both challenge groups (*p* < 0.05), whereas globulin (GLB) was significantly increased in the immersion group compared with control and injection (*p* < 0.05), suggesting a stronger humoral/protein fraction shift in immersion-challenged fish. Finally, the AST/ALT ratio differed among groups, being lower in injection than in control, with immersion showing an intermediate pattern (control > immersion > injection, letter grouping), consistent with the observed elevations in aminotransferases after challenge.

### 2.7. Histopathology Analysis

**Figure 8:**
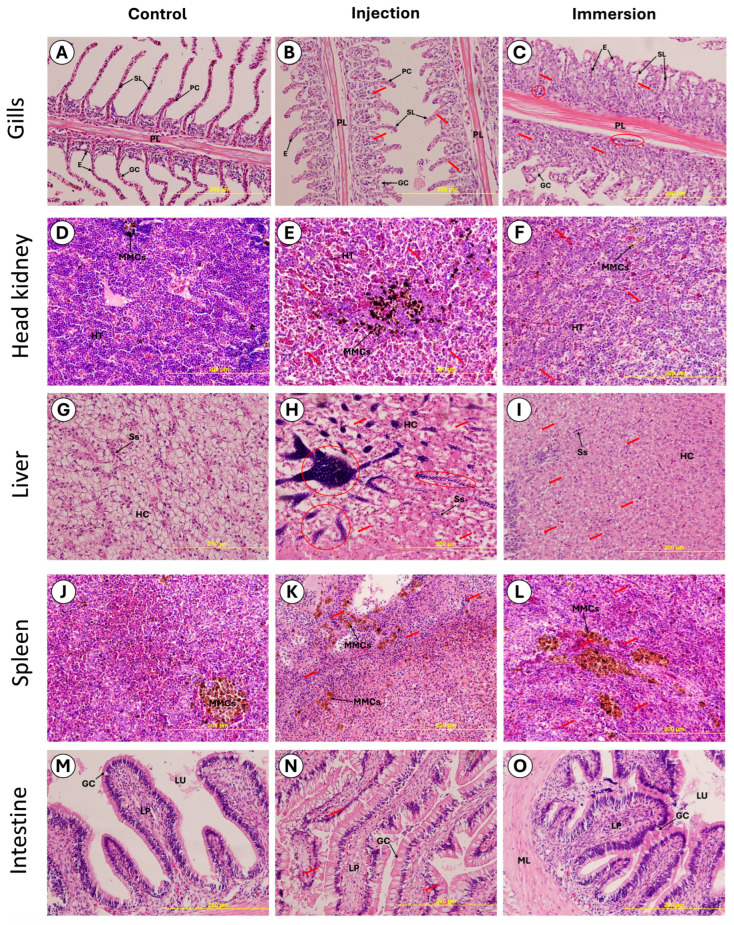
Histopathological changes in Nile tilapia following *L. garvieae* AAHM-LG2501 challenge by injection and immersion (H&E). Representative micrographs of (**A**–**C**) gills, (**D**–**F**) head kidney, (**G**–**I**) liver, (**J**–**L**) spleen, and (**M**–**O**) intestine from control, injection-challenged, and immersion-challenged fish, respectively. Control tissues show normal architecture, whereas challenged groups exhibit multi-organ lesions consistent with systemic bacterial infection. Red arrows indicate representative lesion areas; circled regions highlight focal pathological foci. Abbreviations shown in the figure include erythrocytes (E), primary lamellae (PL), secondary lamellae (SL), pillar cells (PC), gill chloride cells (GCC), hematopoietic tissue (HT), melanomacrophage centers (MMCs), hepatocytes (HCs), sinusoids (Ss), lamina propria (LP), lumen (LU), muscular layer (ML), and goblet cells (GCs). Scale bars = 200 µm.

Across all sampled organs, including the gills, head kidney, liver, spleen, and intestine, control fish showed normal tissue architecture without remarkable inflammatory infiltration, necrotic foci, or vascular alterations. In contrast, histopathological lesions were observed in all challenged fish and involved multiple organs. Overall, lesions were generally milder and more variable following immersion exposure, whereas intraperitoneal injection produced more consistent and severe systemic lesions, particularly in the head kidney, liver, and spleen, supporting the greater systemic impact of the injection route (Figure 8).

#### 2.7.1. Gills

Control fish displayed normal gill architecture with well-organized primary and secondary lamellae and intact epithelial lining. In the injection-challenged group, gills showed clear pathological alterations, including lamellar epithelial lifting/edema and focal hyperplastic changes, with areas suggestive of lamellar fusion and local tissue disruption (arrows). Lesions were also evident in the immersion group and were characterized by more extensive epithelial lifting and thickening of lamellar tissues, consistent with a stronger surface-associated response, together with multifocal structural disturbance along the lamellae (Figure 8A–C).

#### 2.7.2. Head Kidney

Control head kidney sections showed dense, uniform hematopoietic tissue with normal melanomacrophage centers (MMCs). In injection-challenged fish, the head kidney exhibited marked tissue disorganization, with prominent MMC activation/aggregation and multifocal vascular congestion/hemorrhagic changes (arrows), indicating systemic involvement. Immersion-challenged fish also showed MMCs activation and multifocal circulatory disturbance, but the overall lesion pattern appeared more patchy compared with injection, consistent with variable systemic progression after immersion exposure (Figure 8D–F).

#### 2.7.3. Liver

Liver from control fish showed preserved hepatic cord arrangement and normal sinusoidal spaces. In injection-challenged fish, the liver presented multifocal hepatocellular degeneration with focal lesions (circled areas) and sinusoidal congestion, consistent with systemic bacterial effects and hepatocellular stress. In immersion-challenged fish, hepatic changes were also apparent and included diffuse hepatocellular alteration with multifocal congestion (arrows), indicating that immersion exposure was sufficient to induce systemic hepatic pathology, although lesion distribution appeared more diffuse rather than sharply focal (Figure 8G–I).

#### 2.7.4. Spleen

Control spleens showed typical splenic tissue with limited MMC prominence. Both challenge routes induced obvious splenic responses. Injection-challenged fish exhibited pronounced MMC expansion/aggregation with multifocal congestion and cellular disturbance (arrows), consistent with activation of splenic clearance and inflammatory processes. Immersion-challenged fish showed a similar pattern with large MMC aggregates and multifocal circulatory disturbance, supporting strong immune activation following infection (Figure 8J–L).

#### 2.7.5. Intestine

Control intestines displayed intact mucosal architecture with normal lamina propria and visible goblet cells. Injection-challenged fish showed mucosal and lamina propria alterations, including multifocal congestion/hemorrhagic foci (arrows) and mild structural disturbance of the mucosal folds. In immersion-challenged fish, intestinal changes were present but generally appeared milder, with localized mucosal alterations and preserved overall tissue architecture, consistent with secondary intestinal involvement during systemic infection (Figure 8M–O).

## 3. Discussion

This study provides integrated evidence that *Lactococcus garvieae* is a clinically important cause of septicemic disease in cage-cultured Nile tilapia in Thailand, supported by field outbreak observations, culture-based recovery, near full-length 16S rRNA confirmation with phylogenetic placement, tissue-associated microbiome patterns, controlled challenge (LD_50_ and LC_50_), and multi-organ histopathology. Taken together, the findings indicate that *L. garvieae* can circulate efficiently in open-water cage systems, establish systemic infection, and produce dose-dependent mortality and characteristic lesions that align with lactococcosis.

### 3.1. Field Presentation and Outbreak Behavior in Cage Culture

The field case definition captured acute mortality events in multiple cages within the same reservoir area, consistent with outbreak-like behavior typical of shared-water systems. Cage farming concentrates biomass and increases fish-to-fish contact, while hydrological connectivity allows continuous exposure to pathogens present in the water column. These features likely amplify transmission once *L. garvieae* is introduced or becomes dominant under stress [1]. The clinical pattern described, including lethargy, anorexia, darkening, exophthalmia, and abnormal behavior, is compatible with systemic bacterial disease and is difficult to distinguish clinically from streptococcosis or other septicemic syndromes. This overlap supports the need for standardized sampling and laboratory confirmation to avoid misclassification and inappropriate interventions [3,9].

Environmental variability is a major driver in cage systems. Even when average water quality appears acceptable, short-term drops in dissolved oxygen, thermal peaks, crowding, handling, and fluctuating organic loading can compromise mucosal barriers and innate defenses [13]. Such stressors can shift the balance from subclinical carriage or low-level exposure toward overt septicemia. In Thailand, seasonal warming and reservoir stratification can further increase outbreak risk by promoting pathogen growth and stressing fish through reduced oxygen availability, especially during calm periods and high biomass.

### 3.2. Tissue Distribution and the Significance of Blood Dominance by L. garvieae

The full-length 16S rRNA results showed strong tissue partitioning between blood and gills. Blood samples were overwhelmingly dominated by *Lactococcus* at the genus level and by *L. garvieae* at the species level, whereas gill communities were diverse and included multiple environmental and opportunistic taxa. This pattern is biologically consistent with septicemia, where the causative agent becomes dominant in blood due to systemic replication and reduced competing microbiota. In contrast, the gill surface is an interface with continuous microbial immigration from water, so higher richness and mixed community composition are expected even during disease [14].

The consistent enrichment of *L. garvieae* in blood strengthens causality because it reflects infection within a normally low-biomass compartment and reduces the likelihood that the signal reflects external contamination or transient exposure alone. The gill community, while informative for exposure ecology, is less specific because opportunistic bacteria such as *Aeromonas* spp. and other taxa may bloom under stress or tissue damage without being primary drivers. Therefore, combining blood-based microbiome evidence with culture recovery and molecular confirmation provides a robust diagnostic triangulation.

It should be noted, however, that the overwhelming dominance of *L. garvieae* in blood microbiome samples introduces substantial compositional bias, which may distort relative abundance estimates and consequently inflate or suppress alpha-diversity indices while confounding ordination-based beta-diversity analyses. In the present study, diversity metrics and ordination analyses were performed on relative abundance data without prior removal or downweighting of the dominant taxon. Accordingly, the alpha- and beta-diversity patterns observed in blood samples should be interpreted with caution given the extreme compositional skewness introduced by the dominant pathogen. Future studies should consider employing compositional data analysis (CoDA) frameworks, such as centered log-ratio (CLR) transformation, to more rigorously account for such dominance effects.

### 3.3. Isolation and Identification Support Systemic Infection and Guide Diagnostics

Culture-based recovery was markedly more consistent from blood than from gills, which aligns with the sequencing evidence and with the pathogenesis of lactococcosis. Blood sampling is also less affected by surface contamination and mixed flora compared with gill, improving the probability of obtaining a representative isolate. The phenotypic profile of Gram-positive, catalase-negative, oxidase-negative, non-motile cocci further supports lactic acid bacteria characteristics, but phenotyping alone is not sufficient for species-level identification. The phylogenetic clustering of all isolates within the *L. garvieae* clade and separation from *L. petauri* is important because closely related lactococci can be difficult to resolve with limited markers. Although species-level discrimination between *L. garvieae* and the closely related *L. petauri* cannot be reliably achieved using *16S rRNA* gene sequencing alone, owing to the high degree of interspecific sequence similarity within this genomic region, the phylogenetic analysis presented in Figure 5 demonstrated that all 16 study isolates (AAHM-LG2501–AAHM-LG2516; GenBank accession numbers PZ007933–PZ007948) clustered within the *L. garvieae* clade together with reference *L. garvieae* sequences and were clearly separated from a distinct *L. petauri* clade composed of multiple reference strains, thereby reducing the likelihood of misassignment based on 16S rRNA similarity alone. The combined approach, including near full-length 16S rRNA sequencing and phylogenetic placement, provides a practical identification workflow for frontline laboratories [15], although the use of additional molecular markers such as *gyrB*, *rpoB*, MLST, or WGS is recommended in future studies to achieve unambiguous species-level resolution.

A practical implication is that outbreak investigations should prioritize blood for primary isolation and confirmatory molecular testing, while gill sampling remains valuable for understanding concurrent microbial complexity and possible co-infection risk. This is particularly relevant in cage systems where opportunistic bacteria are common and may alter disease severity or treatment outcomes.

### 3.4. Virulence and Pathogenicity Inferred from LD_50_ and LC_50_ Patterns

The controlled challenge results indicate that *L. garvieae* AAHM-LG2501 is virulent to Nile tilapia through both systemic and mucosal exposure routes. AAHM-LG2501, a blood-derived and molecularly confirmed *Lactococcus garvieae* isolate, was selected as a representative outbreak strain to ensure a standardized, reproducible inoculum for reliable LD_50_ and LC_50_ estimation.

The injection LD_50_ of approximately 1.05 × 10^5^ CFU/fish demonstrates that relatively modest doses can produce lethal systemic disease when the pathogen bypasses mucosal barriers. The immersion LC_50_ of approximately 1.20 × 10^6^ CFU/mL indicates that waterborne exposure can also cause substantial mortality, but at higher concentrations, which is expected because immersion requires successful colonization at external surfaces followed by invasion.

The difference between injection and immersion routes provides mechanistic insight. Injection models the outcome once the bacterium gains systemic access, while immersion models natural exposure in cages. In open-water farming, fish are continually exposed to fluctuating pathogen loads, and risk increases when environmental conditions allow pathogen amplification, when mucosal defenses are compromised, or when handling causes micro-injuries. Therefore, the immersion LC_50_ has direct relevance to outbreak prevention because it approximates the environmental intensity needed to trigger high mortality under the experimental conditions.

The blood biochemistry profiles indicate that *L. garvieae* challenge induced selective, organ-linked physiological disturbance rather than broad systemic metabolic collapse, as most chemistry indices remained comparable among control, injection, and immersion groups. The rationale for including a comprehensive blood biochemistry panel in the present study was threefold, serving purposes that extend beyond supplementary confirmation of histopathological findings. First, the biochemistry data provide quantitative, objective evidence of organ dysfunction that cross-validates and extends the histopathological observations. The significant elevations of ALT and AST in both injection and immersion groups confirm hepatocellular injury and/or systemic tissue stress, and importantly demonstrate that the severity of hepatic involvement is functionally significant a dimension that descriptive histopathology alone cannot fully quantify. This supports the interpretation, discussed in the context of the histopathological findings, that hepatic involvement is an early and sensitive component of *L. garvieae* lactococcosis pathophysiology. Second, the biochemistry panel captures route-associated and systemic physiological responses that are not reflected in tissue morphology. The significantly elevated globulin in the immersion-challenged group suggests greater mucosal immune stimulation and antigen exposure via the waterborne route, whereas injection-challenged fish showed a different pattern consistent with rapid systemic damage. The route-dependent AST/ALT ratio pattern (control > immersion > injection) reveals mechanistic differences in tissue response and enzyme release between challenge routes findings that are directly relevant to understanding the pathophysiology of natural cage-culture exposure versus systemic invasion. Third, the significant elevation of amylase in both challenged groups may reflect stress-related pancreatic or intestinal involvement, or leakage of digestive enzymes secondary to systemic inflammation providing evidence of multi-organ involvement beyond what is captured by the five organs selected for histopathological examination. Taken together, the blood biochemistry data complement rather than duplicate the histopathological findings, and their collective interpretation reinforces that lactococcosis primarily manifests as hepatic and inflammatory stress signatures with route-dependent physiological nuances.

### 3.5. Histopathology Reveals Route-Dependent Severity and Systemic Dissemination

Histopathology supported systemic infection with route-dependent lesion severity. Across organs, the injection challenge produced more consistent and severe lesions than immersion, which is expected because systemic inoculation accelerates dissemination. The qualitative nature of the histopathological assessment, as detailed in Section 2.7, was considered appropriate for the confirmatory objectives of the present study, given that the complete absence of pathological changes in control fish and the consistent presence of multi-organ lesions in all challenged fish provided unambiguous group-level discrimination without the requirement for formal lesion scoring or statistical comparison. The head kidney showed the most consistent and severe injury following injection, including congestion, inflammatory infiltration, and disruption of hematopoietic tissue architecture [16]. In teleosts, the head kidney is central to hematopoiesis and immune function, and severe lesions there are consistent with septicemia and dysregulated immune responses. The liver and spleen also showed prominent congestion and inflammatory changes, aligning with systemic bacterial circulation, immune activation, and vascular injury [17].

Gill lesions were present in both routes but were more pronounced after injection, suggesting that gill pathology may reflect both direct exposure effects and secondary systemic consequences such as vascular congestion and inflammatory trafficking. The intestine showed clearer lesions after injection, consistent with systemic disease effects rather than primary mucosal exposure. This supports a model in which severe systemic infection leads to multi-organ vascular compromise and inflammatory responses, which can manifest in tissues not directly exposed to bacteria in the immersion period. Overall, these lesion patterns are consistent with septicemic disease biology and provide tissue targets that can be prioritized for routine histopathological confirmation during outbreak investigations, particularly head kidney, spleen, and liver.

### 3.6. Role of Mixed Gill Microbiota and Potential Co-Infections

The gill microbiome in challenged fish included multiple taxa often associated with opportunism or environmental exposure, such as *Aeromonas* spp. and other genera. This does not necessarily indicate that these taxa were primary pathogens in the cases studied, but it does suggest a biologically plausible pathway for co-infections or secondary infections that could intensify disease severity [18]. In cage systems, fish experiencing *L. garvieae* septicemia may have compromised barriers and impaired immunity, allowing other bacteria to invade or exacerbate inflammatory damage. From a management perspective, this supports the use of multiplex diagnostic panels, especially during severe outbreaks, to identify mixed infections that could influence treatment decisions and mortality patterns.

### 3.7. Implications for Surveillance and Outbreak Investigation in Thailand

This study highlights practical actions to strengthen lactococcosis surveillance and outbreak investigations in Thailand’s cage-cultured tilapia systems: blood should be prioritized for bacteriology and molecular confirmation because it offers higher diagnostic specificity and more consistent isolation success, while gill samples can be collected alongside to provide exposure context and potential co-infection signals. Confirmatory identification is best achieved using near full-length 16S rRNA sequencing with phylogenetic placement, as demonstrated in the present study whereby all isolates clustered within the *L. garvieae* clade and were clearly separated from the *L. petauri* clade, thereby reducing the likelihood of misassignment based on 16S rRNA similarity alone [19]. However, the isolates were identified based on conventional bacteriological characterization in combination with *16S rRNA* gene sequencing and phylogenetic analysis. However, because no species-specific PCR assay or additional housekeeping gene markers were included, species-level discrimination from closely related taxa, particularly *Lactococcus petauri*, should be interpreted with caution. Farm-level mortality thresholds remain useful for triggering rapid response, but they should be coupled with immediate diagnostic sampling to minimize delays. Finally, routine recording of key environmental variables (temperature, dissolved oxygen, pH, transparency) should be integrated with mortality trends and management data (biomass, feeding intensity, handling events), with particular attention given to temperature spikes and oxygen drops as early warning triggers for intensified monitoring.

From a forward-looking perspective, lactococcosis in cage-cultured tilapia is likely to remain a recurrent and potentially escalating threat as multiple risk factors converge. Warmer water can accelerate *Lactococcus garvieae* proliferation while simultaneously increasing fish metabolic demand and reducing oxygen availability, together heightening stress and shortening the interval between exposure and mortality. Intensification of production, including higher stocking densities, larger cage clusters, and faster cycles, further increases contact rates and pathogen amplification, enabling rapid spread once the bacterium is introduced. In connected reservoir and river systems, bacteria can be transported through shared water, and transmission can be reinforced by farm practices such as shared boats, nets, and personnel when biosecurity is inconsistent. Compounding this, *L. garvieae* may persist at low levels in fish or the environment and emerge as clinical disease mainly during stress or after introduction of more virulent strains, limiting the effectiveness of surveillance based only on visible signs. Finally, co-infections and opportunistic microbes common in cage environments can intensify disease severity, turning otherwise moderate infections into major mortality events when fish immunity is compromised.

Future studies should (1) integrate environmental time series and farm management data for spatiotemporal risk modeling, (2) apply whole-genome sequencing to resolve strain diversity, virulence profiles, and transmission routes, (3) quantify co-infections via multiplex PCR or metagenomics to explain variability in lesions and mortality, and (4) evaluate immunostimulant agents, probiotics and vaccines under cage-relevant conditions to support practical control [7,20,21,22,23,24,25]. Overall, the evidence indicates that *L. garvieae* is a major driver of septicemic outbreaks in cage-cultured tilapia in Thailand, supported by its consistent detection and isolation from blood, dose-dependent lethality via injection and immersion, and systemic pathology. Although risk may rise with warming and production intensification, improved surveillance, stronger biosecurity, and immunoprophylaxis offer feasible strategies to reduce impacts.

## 4. Materials and Methods

### 4.1. Ethical Statement

The experimental procedures involving aquatic animals were conducted in accordance with the Ethical Principles and Guidelines for the Use of Animals outlined by the National Research Council of Thailand, which governs the care and use of animals for research purposes. This protocol received approval from the Animal Ethics Committee at Kasetsart University in Thailand (Approval ID: ACKU68-CSC-004, 25 July 2025).

### 4.2. Study Area, Case Definition, Sampling Frame and Environmental Metadata Analysis

Sampling was carried out from May to October 2025 during mortality events in cage-cultured Nile tilapia (*Oreochromis niloticus*) reared in floating net cages in a reservoir located in Ubon Ratchathani Province, Thailand. The cages measured 5 m × 5 m with a depth of 2.5 m and were stocked at approximately 1000 fish per unit. The sampled fish ranged in body weight from 200 to 900 g.

A suspected case was defined as any cage experiencing an abrupt increase in daily mortality greater than 5% over one or more days, with affected fish showing lethargy, anorexia, darkening, exophthalmia, or neurologic signs. A confirmed case required laboratory detection of *Lactococcus garvieae* from blood and gill tissues, which were defined based on bacteriological findings together with molecular identification by *16S rRNA* gene sequencing.

#### Environmental Metadata During Mortality Events

During each sampling visit, key environmental parameters were recorded to contextu-alize mortality events and facilitate comparisons among outbreaks. Water temperatures (morning and evening) were measured using thermometer, dissolved oxygen (DO), pH, total alkalinity, and ammonia (NH_3_) were measured using drop-count titration and colorimetric test kits (PARA Test, Bangkok, Thailand), and total hardness using the Hanna Instruments HI3812 test kit (Hanna Instruments, Smithfield, RI, USA). Total dissolved solids (TDS), salinity, and electrical conductivity were measured using the YSI Pro Quatro multiparameter instrument model no. 606963 and YSI Pro Digital instrument model no. 626972 (YSI Incorporated, Yellow Springs, OH, USA). All measurements were performed according to the respective manufacturer’s instructions, and multiparameter instruments were calibrated prior to each field sampling session following the manufacturer’s recommended procedures.

### 4.3. Tissue Distribution of the Causative Bacterium in Blood and Gills of Infected Fish from Cage-Cultured Tilapia Identified by Full-Length 16S rRNA Sequencing

#### 4.3.1. Fish Sampling and Tissue Collection

Eight moribund tilapia showing clinical signs consistent with bacterial septicemia were collected from cage-culture sites in Thailand. Fish were euthanized following institutional animal welfare guidelines. External surfaces were wiped with 70% ethanol prior to dissection. Using sterile instruments, blood was collected aseptically from the caudal vein into sterile microtubes. Gill tissues were excised from the first gill arch using sterile scissors and forceps. Each specimen was placed into sterile DNA-free tubes, kept on ice during transport, and stored at −80 °C until DNA extraction. To minimize cross-contamination, instruments were changed or flame-sterilized and rinsed with 70% ethanol between fish and between tissue types.

#### 4.3.2. DNA Extraction and Contamination Controls

Microbial DNA was extracted from (i) whole blood and (ii) gill tissue homogenates using the ZymoBIOMICS^TM^ DNA Miniprep Kit (Zymo Research, Irvine, CA, USA), following the manufacturer’s instructions with minor modifications. DNA quantity and purity were assessed by spectrophotometry (A260/280 and A260/230) and fluorometry. DNA integrity was checked by agarose gel electrophoresis. All extracts were stored at −20 °C.

Given that blood is a low-biomass sample type with inherent susceptibility to exogenous contamination, rigorous contamination controls were implemented throughout the DNA extraction and library preparation workflow. Extraction blank controls, consisting of nuclease-free water processed through the complete ZymoBIOMICS™ DNA Miniprep Kit extraction protocol in parallel with each sample batch, were included in every extraction run.

Sequence-based identification was performed using full-length *16S rRNA* gene sequencing on the PacBio SMRT platform. The full-length *16S rRNA* gene was amplified using barcoded universal primers targeting conserved regions flanking the entire gene, namely 27F (5′-AGRGTTTGATYNTGGCTCAG-3′) and 1492R (5′-TASGGHTACCTTGTTASGACTT-3′). Sequence data were processed using the BMKGENE bioinformatics pipeline (Amplicon Sequencing Data Analysis Method, version 2.7). Raw reads were quality-filtered using Trimmomatic (v0.40, sliding window 50 bp; average Q-score cutoff 20), primers were removed with Cutadapt (v5.2, max mismatch 20%; minimum coverage 80%), paired-end reads were merged using USEARCH (v10, minimum overlap 10 bp; minimum overlap similarity 90%), and chimeras were removed using UCHIME (v12.0) to obtain high-quality clean reads for downstream analyses. No-template PCR controls were additionally included in every amplification batch. Sequencing reads generated from negative controls were cross-referenced with the corresponding sample dataset, and any ASVs detected in negative controls at relative abundances equal to or exceeding those observed in the corresponding samples were flagged and excluded from downstream diversity analyses and taxonomic profiling.

Microbial features were analyzed using an OTU-based approach. Operational taxonomic units (OTUs) were clustered at 97% sequence similarity using USEARCH (v10), and low-abundance features were filtered using a conservative 0.005% threshold. Taxonomic assignment was performed using the SILVA database (Release 138) for *16S rRNA* gene annotation.

Alpha diversity (Chao1, ACE, Shannon, Simpson, and phylogenetic diversity) was calculated in QIIME2, and beta diversity was evaluated using distance metrics including Bray–Curtis, Jaccard, and weighted/unweighted UniFrac, followed by ordination analyses (PCA, PCoA, and NMDS). Group differences in community structure were assessed using ANOSIM and Adonis (permutational MANOVA) based on the distance matrices.

### 4.4. Isolation of the Causative Bacterium in Blood and Gills of Infected Fish from Cage-Cultured Tilapia

#### 4.4.1. Isolation, Culture Conditions and Phenotypic Analyses of *Lactococcus garvieae*

Primary isolation focused on Gram-positive cocci consistent with *Lactococcus* spp. Aseptically collected tissues and whole blood were streaked onto tryptic soy agar (TSA), brain heart infusion agar (BHIA), de Man, Rogosa and Sharpe (MRS) agar, and blood agar prepared on a tryptic soy base supplemented with 5% sheep blood (TSBA). Plates were incubated at 28 to 30 °C for 24 to 48 h. Colony morphology was recorded, and representative colonies were re-streaked until pure cultures were obtained.

Presumptive isolates were characterized using Gram staining and basic phenotypic tests, including hemolysis pattern on TSBA, catalase and oxidase reactions, and motility assessment, following standard protocols for fish bacterial pathogens and lactic acid bacteria. A limited biochemical panel appropriate for lactic acid bacteria was used for preliminary grouping, but isolates were assigned to species only after molecular confirmation. For long-term preservation, pure cultures were stored at −80 °C in tryptic soy broth (TSB) supplemented with 30% (*v*/*v*) glycerol.

#### 4.4.2. Molecular Identification Full-Length 16S rRNA Sequencing of Isolates

Bacterial isolates were homogenized for bacterial DNA extraction using the ZymoBIOMICS^TM^ DNA Miniprep Kit (Zymo Research, USA), following the manufacturer’s instructions. DNA concentration and purity were verified using a NanoDropTM spectrophotometer (Thermo Fisher 326 Scientific, Waltham, MA, USA).

The near full-length *16S rRNA* gene was amplified using universal primers 27F (5′-AGAGTTTGATCMTGGCTCAG-3′) and 1492R (5′-TACGGYTACCTTGTTACGACTT-3′). PCR was performed in a 25 µL reaction containing 1× high-fidelity buffer, 0.2 mM each dNTP, 0.4 µM of each primer, 1.25 U high-fidelity DNA polymerase, and 1 µL of template DNA. Cycling conditions were 95 °C for 3 min, followed by 30 cycles of 95 °C for 30 s, 55 °C for 30 s, and 72 °C for 90 s, with a final extension at 72 °C for 5 min. Amplicons of approximately 1450 bp were confirmed on 1.0% agarose gel, purified, and Sanger sequenced bidirectionally. Forward and reverse reads were assembled to generate consensus sequences and compared against curated reference databases for initial genus-level assignment. Only high-quality chromatograms were retained; samples with ambiguous base calls were re-amplified and re-sequenced or excluded. All sixteen full-length *16S rRNA* gene sequences generated in this study have been deposited in the NCBI GenBank database under accession numbers PZ007933–PZ007948 (isolates AAHM-LG2501 to AAHM-LG2516, respectively).

For phylogenetic confirmation, the study sequences and closely related reference *16S rRNA* sequences were aligned using MUSCLE. The alignment was then imported into MEGA (Molecular Evolutionary Genetics Analysis) to infer phylogenetic relationships. A phylogenetic tree was constructed using the neighbor-joining method with 1000 bootstrap replicates to evaluate node support. The resulting topology was used to confirm clustering of the isolates within the *Lactococcus* clade and to visualize relationships with closely related taxa.

### 4.5. Controlled Exposure Model for Pathogenicity Assessment

#### 4.5.1. Bacterial Strain and Inoculum Preparation

Among the sixteen isolates, strain identity was confirmed, and a well-characterized *Lactococcus garvieae* strain AAHM-LG2501, isolated from the blood of diseased Nile tilapia collected from the field, was selected for the challenge test. The bacterium was initially revived from −80 °C stocks on tryptic soy agar supplemented with 5% sheep blood and incubated at 28–30 °C for 24 h. A single colony was then inoculated into tryptic soy broth and cultured at 28–30 °C with shaking at 180 rpm to mid-log phase (OD_600_ ≈ 0.6–0.7). Cells were harvested, washed twice with sterile phosphate-buffered saline (PBS), and the concentration was adjusted spectrophotometrically using a pre-established OD_600_–CFU standard curve. Triplicate spread plating confirmed the OD600–CFU relationship, with an OD600 of 1.0 corresponding to 1.0 × 10^9^ CFU/mL.

#### 4.5.2. Experimental Fish and Husbandry

Healthy Nile tilapia were obtained from the hatchery of the Faculty of Fisheries, Kasetsart University, Thailand, and acclimated for 14 days. Fish (200–250 g) were used (adult stage). Fish were clinically healthy with no antibiotic exposure for at least 4 weeks before the trial. Experiments were conducted in aerated 250 L glass tanks at 27–29 °C, dissolved oxygen ≥ 5 mg/L, pH 7.0–7.8, and a 12 h light:12 h dark photoperiod. Fish were fed 4.0% body weight per day and were fasted 24 h before challenge. Each treatment had three independent tank replicates with 20 fish per tank.

#### 4.5.3. LC_50_ (Immersion) and LD_50_ (Injection) Screening

(1) Immersion challenge procedure

The median lethal concentration (LC_50_; CFU/mL) was estimated using an immersion exposure model. For each immersion tank, the water volume was adjusted to 40 L, and the bacterial inoculum was added to achieve final concentrations of 10^1^, 10^2^, 10^3^, 10^4^, 10^5^, 10^6^, 10^7^, 10^8^ CFU/mL. Fish were exposed for 60 min with vigorous aeration. Sham control tanks received an equivalent volume of clean water. After exposure, fish were transferred to new 250 L tanks containing conditioned, aerated water, and no water exchange was performed during the first 24 h post-challenge. Each treatment included three independent tank replicates, with 20 fish per tank.

(2) Injection challenge procedure

The median lethal dose (LD_50_; CFU/fish) was estimated following intraperitoneal injection to bacterial virulence via a systemic route. Fish were injected with 0.1 mL per fish containing 10^1^, 10^2^, 10^3^, 10^4^, 10^5^, 10^6^, 10^7^, 10^8^ CFU/fish under approved anesthesia. Sham controls received 0.1 mL of sterile phosphate-buffered saline (PBS). Injection sites were disinfected prior to dosing. Injected groups were maintained and monitored under the same husbandry conditions as the immersion-challenged groups. Each treatment included three independent tank replicates, with 20 fish per tank.

(3) Clinical monitoring, endpoints, and sampling

Fish were observed at least twice daily for 14 days for clinical signs and mortality. Humane endpoints were predefined as severe lethargy, loss of equilibrium, persistent surface piping, or non-responsive behavior. Moribund fish and all dead fish were necropsied and sampled for bacteriology, molecular confirmation, and histopathology. Gills, head kidney, liver, spleen, and intestine were collected aseptically. Whole blood was also collected to measure blood biochemistry analysis. Survivors at the study’s end were humanely euthanized and sampled similarly.

(4) Histopathology

Formalin-fixed tissues from the challenge test from control immersion and injection, including gills, head kidney, liver, spleen, and intestine, were processed to paraffin, sectioned at 4–5 µm, and stained with hematoxylin and eosin.

(5) Blood biochemistry analysis

Serum was analyzed using a Pushkang MSC100V veterinary coagulation and chemistry analyzer (Zhejiang Pushkang Biotechnology Co., Ltd., Shaoxing, China) with the veterinary general chemistry 23-analyte panel (cat. no. VE60010). Samples were processed immediately for the following parameters: albumin (ALB), total protein (TP), alkaline phosphatase (ALP), alanine aminotransferase (ALT), aspartate aminotransferase (AST), gamma-glutamyl transferase (GGT), direct bilirubin (DBIL), total bilirubin (TBIL), indirect bilirubin (IBIL), globulin (GLB), urea, creatinine (CREA), calcium (Ca), inorganic phosphorus (P), total carbon dioxide (tCO_2_), glucose (GLU), cholesterol, amylase (AMY), creatine kinase (CK), total bile acids (TBAs), albumin/globulin ratio (ALB/GLB), urea/creatinine ratio (UREA/CREA), and AST/ALT ratio.

(6) Statistical data analysis

All statistical analyses were performed using GraphPad Prism version 10.4.0 for MacOSX (GraphPad Software, Boston, MA, USA). The median lethal concentration (LC_50_) and median lethal dose (LD_50_) were estimated from cumulative mortality data using dose–response analysis with probit regression on log10-transformed dose/concentration. LC_50_/LD_50_ values and their 95% confidence intervals were defined as the dose/concentration corresponding to 50% predicted mortality, and model fit was evaluated by comparing observed and predicted mortality patterns.

Blood biochemistry data were analyzed by one-way analysis of variance (ANOVA) followed by Tukey’s multiple-comparison test. Data are presented as mean ± SD. Differences were considered statistically significant at *p* < 0.05.

## 5. Conclusions

This study confirms *L. garvieae* as a major cause of acute septicemic disease in cage-cultured Nile tilapia in Thailand, supported by field investigations, culture isolation, full-length 16S rRNA profiling with phylogenetic confirmation, challenge trials, and histopathology. Blood consistently showed dominance of *L. garvieae* and yielded isolates more reliably than gill tissue, indicating blood is the most informative specimen for frontline diagnosis. Pathogenicity testing demonstrated dose-dependent mortality via both routes, with an LD_50_ of ~1.05 × 10^5^ CFU/fish (intraperitoneal injection) and an LC_50_ of ~1.20 × 10^6^ CFU/mL (immersion), confirming lethality through systemic and waterborne exposure. Histopathology supported systemic dissemination, with injection producing more consistent and severe multi-organ lesions than immersion, particularly in the head kidney, liver, and spleen. Collectively, the workflow provides a practical framework for outbreak surveillance and highlights the need for risk-based monitoring, stronger biosecurity, and preventive measures such as vaccination to reduce lactococcosis impacts in intensive cage farming.

## Data Availability

The near full-length *16S rRNA* gene sequences generated in this study have been deposited in the NCBI GenBank database under accession numbers PZ007933–PZ007948 (isolates AAHM-LG2501 to AAHM-LG2516, respectively). Other data supporting the findings of this study are available from the corresponding authors upon reasonable request.
